# Ovariectomy-Induced Dysbiosis May Have a Minor Effect on Bone in Mice

**DOI:** 10.3390/microorganisms9122563

**Published:** 2021-12-10

**Authors:** Satoshi Kosaka, Yuji Nadatani, Akira Higashimori, Koji Otani, Kosuke Fujimoto, Yuki Nagata, Masaki Ominami, Shusei Fukunaga, Shuhei Hosomi, Noriko Kamata, Fumio Tanaka, Yasuaki Nagami, Koichi Taira, Seiya Imoto, Satoshi Uematsu, Toshio Watanabe, Yasuhiro Fujiwara

**Affiliations:** 1 Department of Gastroenterology, Osaka City University Graduate School of Medicine, Osaka 545-8585, Japan; m2049012@med.osaka-cu.ac.jp (S.K.); higamo@med.osaka-cu.ac.jp (A.H.); kojiotani@med.osaka-cu.ac.jp (K.O.); ominami@med.osaka-cu.ac.jp (M.O.); m1156849@med.osaka-cu.ac.jp (S.F.); hosomi.shuhei@med.osaka-cu.ac.jp (S.H.); nkamata@med.osaka-cu.ac.jp (N.K.); m2079981@med.osaka-cu.ac.jp (F.T.); yasuaki-75@med.osaka-cu.ac.jp (Y.N.); koichit@med.osaka-cu.ac.jp (K.T.); yasu@med.osaka-cu.ac.jp (Y.F.); 2 Department of Premier Preventive Medicine, Osaka City University Graduate School of Medicine, Osaka 545-8585, Japan; watanabet@med.osaka-cu.ac.jp; 3 Department of Immunology and Genomics, Osaka City University Graduate School of Medicine, Osaka 545-8585, Japan; fujimoto.kosuke@med.osaka-cu.ac.jp (K.F.); uematsu.satoshi@med.osaka-cu.ac.jp (S.U.); 4 Division of Metagenome Medicine, Human Genome Center, The Institute of Medical Science, The University of Tokyo, Minato-ku, Tokyo 108-8639, Japan; 5 Division of Innate Immune Regulation, International Research and Development Center for Mucosal Vaccines, The Institute of Medical Science, The University of Tokyo, Tokyo 108-8639, Japan; 6 Department of Vascular Medicine, Vascular Science Center for Translational Research, Osaka City University Graduate School of Medicine, Osaka 545-8585, Japan; m1112473@med.osaka-cu.ac.jp; 7 Division of Health Medical Intelligence, Human Genome Center, The Institute of Medical Science, The University of Tokyo, Minato-ku, Tokyo 108-8639, Japan; imoto@ims.u-tokyo.ac.jp; 8 Collaborative Research Institute for Innovative Microbiology, The University of Tokyo, Bunkyo-ku, Tokyo 113-8657, Japan

**Keywords:** dysbiosis, osteoporosis, microbiota, fecal microbiota transplantation

## Abstract

We determined the bone mineral density (BMD) and the expression of serum bone formation marker (procollagen type I *N*-terminal propeptide: PINP) and bone resorption marker (*C*-terminal telopeptide of collagen: CTX) by ELISA to evaluate ovariectomy-induced osteoporosis in ovariectomized (OVX) mice. The intestinal microbiota of the mice was assessed using 16S rRNA gene sequencing. OVX mice exhibited a lower BMD of 87% with higher serum levels of CTX and PINP compared to sham-operated (sham) mice. The cecum microbiome of OVX mice showed lower bacterial diversity than that of sham mice. TNFα mRNA levels in the colon were 1.6 times higher, and zonula occludens-1 mRNA and protein expression were lower in OVX mice than in sham mice, suggesting that ovariectomy induced inflammation and increased intestinal permeability. Next, we used antibiotic treatment followed by fecal microbiota transplantation (FMT) to remodel the gut microbiota in the OVX mice. A decrease in PINP was observed in antibiotic-treated mice, while there was no change in BMD or CTX between mice with and without antibiotic treatment. Oral transplantation of the luminal cecal content of OVX or sham mice to antibiotic-treated mice did not affect the BMD or PINP and CTX expression. Additionally, transplantation of the luminal contents of OVX or sham mice to antibiotic-treated OVX mice had similar effects on BMD, PINP, and CTX. In conclusion, although ovariectomy induces dysbiosis in the colon, the changes in the gut microbiota may only have a minor role in ovariectomy-induced osteoporosis.

## 1. Introduction

Osteoporosis is characterized by decreased bone mass and microarchitectural disruption and is the most common bone metabolic disease. Women are at increased risk for osteoporosis than men primarily due to the decline in estrogen levels after menopause [1]. Other factors include a lower amount of bone mass and the inadequate consumption of nutrients during pregnancy. Bone mass is maintained by the balance between osteoblast formation and bone resorption by osteoclasts. Estrogen deficiency causes uncoupled bone remodeling, leading to bone loss. This could be attributed to estrogen’s role in inhibiting osteoclast differentiation and promoting osteoclast apoptosis while inhibiting apoptosis of osteoblasts [2]. Further, inflammation induces osteoclast differentiation and causes excessive bone resorption [3], suggesting that systemic diseases can affect bone mass.

Although many studies have shown a relationship between the gut microbiota and non-intestinal [4,5,6,7] and intestinal diseases [8,9,10], few studies have reported the relationship between bone homeostasis and the gut microbiota [11,12,13,14,15,16]. Recent studies have indicated that gut microbiota composition is altered in patients with osteoporosis [13], but it is not clear whether the changes in the gut microbiota modulate bone homeostasis. It was reported that the germ-free mice had a higher bone mass, which decreased after gut microbiota colonization [11,17]. Ovariectomized rodents that mimic postmenopausal osteoporosis showed reduced bone loss after treatment with probiotics [14,15,18,19]. Additionally, it has been suggested that the gut microbiota regulate estrogens via enterohepatic recirculation [20]. These studies suggest that the gut microbiome may affect bone mass.

In the present study, we aimed to clarify the role of the gut microbiota in bone homeostasis of ovariectomized mice.

## 2. Materials and Methods

### 2.1. Animals

Female C57BL/6J mice were purchased from Charles River Japan (Atsugi, Japan), and specific pathogen-free mice were used in this study. All animals were housed in polycarbonate cages with paper chip bedding in an air-conditioned room with a 12-h light–dark cycle. All animals had free access to food and water.

All experiments were carried out under the control of the Animal Research Committee following the Guidelines on Animal Experiments of Osaka City University Graduate School of Medicine, the Japanese Government Animal Protection and Management Law (No. 105), and the Japanese Government Notification on Feeding and Safekeeping of Animals (No. 6). The Animal Care Committee of the Osaka City University Graduate School of Medicine approved all experimental procedures (approval number 18032). All surgeries were performed under isoflurane, with maximum effort taken to minimize suffering.

### 2.2. Ovariectomized Mice Model

Eight-week-old C57BL/6J female mice were ovariectomized (OVX) or sham-operated (N = 5–10 per group) to evaluate estrogen-deficiency-induced osteoporosis, as previously described [21]. Briefly, a midline dorsal incision was made along the lumbar vertebrae, and the muscle on the flanks was cut to access the abdomen. The ovary, oviduct, and part of the uterus were removed through the incision and resected. The body weight of each group was measured weekly. The mice were sacrificed four weeks after surgery, and the blood, tissue samples from the jejunum and ascending colon, luminal contents of the jejunum and cecum, and left tibia were collected. The tibia was stored in 70% ethanol until further evaluation.

### 2.3. Antibiotic Treatment

We investigated the role of commensal bacteria in osteoporosis with eight-week-old C57BL/6J female mice that were administered a mixture of antibiotics for four weeks: ampicillin (1 g/L; FUJIFILM Wako Pure Chemical Corp., Osaka, Japan), neomycin (1 g/L; FUJIFILM Wako Pure Chemical Corp.), metronidazole (500 mg/L; FUJIFILM Wako Pure Chemical Corp.), and vancomycin (500 mg/L; FUJIFILM Wako Pure Chemical Corp.) mixed in drinking water, to decontaminate the digestive tract (n = 7–10 per group). The control mice received normal drinking water. After the experimental period, blood and the left tibia were collected to assess bone homeostasis.

### 2.4. Transplantation of Cecal Luminal Contents

Fecal microbiota transplantation (FMT) was performed from OVX mice to wild-type mice (N = 8–9) to elucidate the characteristics of commensal bacteria after OVX. Cecal luminal contents were collected from OVX and sham mice. The collected contents were stored at −80 °C until transplantation and suspended in phosphate-buffered saline (PBS; FUJIFILM Wako Pure Chemical Corp.) just before transplantation. Four-week-old C57BL/6J female mice were treated with a mixture of antibiotics, as described above for four weeks, and then were orally administered the prepared suspension three times a week for four weeks (Figure S1A).

Additionally, FMT was performed on antibiotic-treated OVX mice to clarify the effect of the normal bacterial flora on OVX mice (N = 6–7). Mice underwent ovariectomy or a sham operation after antibiotic treatment, and then the recipient mice were administered the prepared in suspension three times a week for four weeks (Figure S1B).

After FMT, the mice were sacrificed, and blood and tissue samples of the colon and left tibia were collected to evaluate intestinal inflammation and bone homeostasis. The experiment was repeated twice.

### 2.5. Micro-Computed Tomography Measurements

Micro-computed tomography (CT) analyses were performed on the proximal tibia using a LaTheta LCT-200 CT scanner (Hitachi Aloka Medical Ltd., Tokyo, Japan) to assess bone mineral density (BMD). The pixel size was 48 µm, and the slice thickness was 96 µm. We analyzed the 1440 μm region beginning at the edge of the proximal growth plate and extending in a distal direction.

### 2.6. Serum Biomarkers of Bone Metabolism

Rodent-specific ELISAs were used to measure serum *C*-terminal telopeptide of collagen (CTX) as a bone resorption marker and serum procollagen type I *N*-terminal propeptide (PINP) as a bone formation marker (AC-06F1 and AC-33F1, Immunodiagnostic Systems, Tyne and Wear, Boldon Colliery, UK) following the manufacturer’s protocol.

### 2.7. Serum Lipopolysaccharide (LPS) Measurement

Serum LPS was measured using mouse LPS ELISA (CSB-E13066m, CUSABIO, Houston, TX, USA) following the manufacturer’s protocol.

### 2.8. 16S rRNA Gene Sequencing for Microbial Analysis

The intestinal luminal samples were homogenized in Tris-EDTA buffer, and bacterial DNA was extracted enzymatically [22]. The 16S rRNA V3–V4 region was amplified by PCR and purified as previously described [22,23]. Microbiota profiles were evaluated by 16S rRNA gene analysis using MiSeq (Illumina, Inc., San Diego, CA, USA) and QIIME2 (version 2018.11; https://qiime2.org; accessed on 20 October 2021), as previously described [24]. Briefly, raw sequence data were subjected to primer sequence trimming, quality filtering, and paired-end read merging using the dada2 denoise-paired method (--p-trim-left-f 17 –p-trim-left-r 21 –p-trunc-len-f 275 –p-trunc-len-r 215 –p-n-threads 4) [25]. Alpha and beta diversity analyses were performed using QIIME diversity core-metrics-phylogenetics based on rarefied sample sequences.

### 2.9. RNA Isolation and Quantitative Reverse Transcription (qRT-)PCR

Total RNA was prepared from the jejunum and ascending colon using an ISOGEN II kit (Nippon Gene Co., Ltd., Tokyo, Japan) following the manufacturer’s protocol. RNA was reverse transcribed into complementary DNA using a High-Capacity RNA-to-cDNA Kit (Thermo Fisher Scientific, Inc., Waltham, MA, USA). qRT-PCR analyses were performed using an Applied Biosystems 7500 Fast Real-Time PCR system (Thermo Fisher Scientific, Inc.). The abundance of mRNA of each gene was standardized to the levels of TaqMan glyceraldehyde-3-phosphate dehydrogenase (GAPDH; Thermo Fisher Scientific Inc.). The primers and probes used for qRT-PCR are listed in Table S1. Additionally, the protein expression level of zonula occludens (ZO)-1 in the jejunum and ascending colon was quantified by western blotting. The expression level of ZO-1 (61–7300, Invitrogen, Carlsbad, CA, USA) was normalized to that of β-actin. The detailed procedure has previously been described [26].

### 2.10. Statistical Analyses

All results are expressed as the mean ± standard error. Welch’s *t*-test was used to compare body weights, BMDs, and mRNA and protein expression levels. The Mann–Whitney U test was used to compare the relative bacterial abundances and bacterial diversities. Permutational multivariate analysis of variance (PERMANOVA) was used to compare bacterial beta diversities. Statistical significance was set at *p* < 0.05. All statistical analyses were performed with R ver. 3.6.2 (The R Foundation for Statistical Computing, https://www.r-project.org/; accessed on 20 October 2021).

## 3. Results

### 3.1. Ovariectomy Decreased Bone Mineral Density

**Figure 1:**
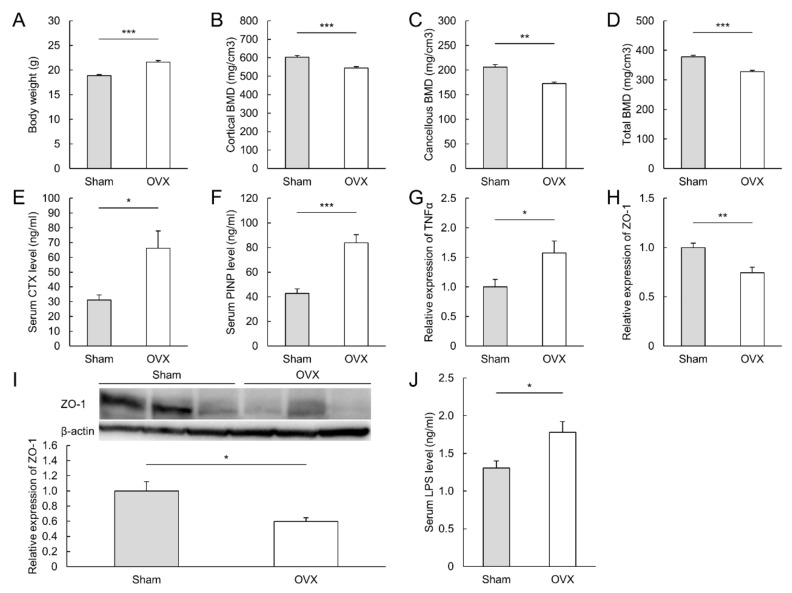
Bone loss and impaired colonic barrier integrity following ovariectomy. (**A**–**F**): Body weight, tibial bone mineral density (**B**–**D**), serum bone formation marker (**E**), and serum bone resorption marker (**F**) at sacrifice. (**G**); The relative expression of TNFα mRNA in colon. (**H**–**J**): Evaluation of colonic barrier integrity. The relative expression of ZO-1 mRNA (**H**) and ZO-1 protein (**I**) in the colon and serum LPS levels (**J**). The protein expression level of ZO-1 was normalized to that of β-actin. N = 5–10 per group. BMD: bone mineral density, CTX: *C*-terminal telopeptide of collagen, LPS: lipopolysaccharide, PINP: procollagen type I *N*-terminal propeptide, TNFα: tumor necrosis factor alpha, ZO-1: zonula occludens-1. * *p* < 0.05, ** *p* < 0.01 and *** *p* < 0.001.

Ovariectomy increased body weight by 15% (Figure 1A) and decreased the cortical, cancellous, and total BMD of the tibia (Figure 1B–D) to 90%, 84%, and 87%, respectively. Evaluation of bone resorption and formation, as indicated by serum CTX-1 (Figure 1E) and PINP (Figure 1F), revealed high turnover in OVX mice.

### 3.2. Ovariectomy Increased the Level of Tumor Necrosis Factor Alpha mRNA and Altered Barrier Function in the Colon

Neither macroscopic nor microscopic signs of inflammation were observed (data not shown). However, in the colon, the expression level of TNFα mRNA in OVX mice was 1.6 times higher than that in sham mice (Figure 1G). Ovariectomy decreased the expression of ZO-1 mRNA in the colon by 74% (Figure 1H), and this decreased expression was also confirmed by western blotting (Figure 1I). The mRNA levels of TNFα and ZO-1 did not change in the small intestine (Figure S2A,B). Furthermore, serum LPS levels were mildly elevated in OVX mice (Figure 1J).

### 3.3. Ovariectomy Decreased Alpha Diversity in the Colon and Altered the Colonic Microbiota

**Figure 2:**
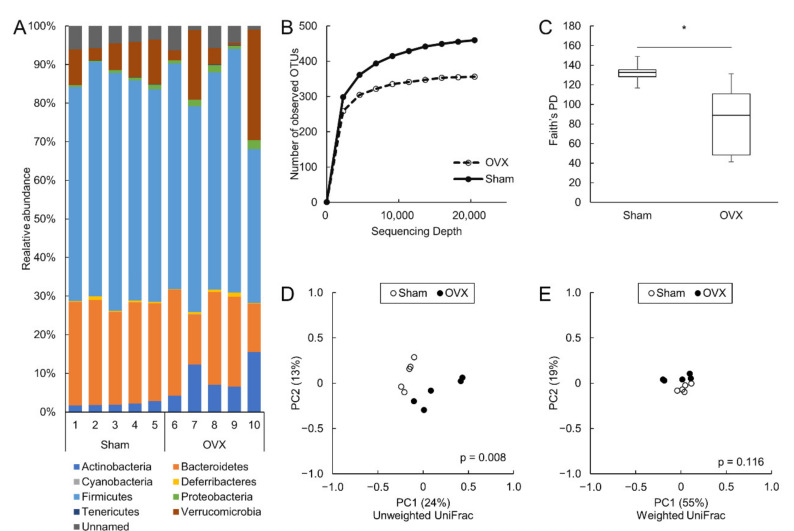
Dysbiosis in colon induced by ovariectomy. (**A**): Colonic microbial composition at the phylum level. (**B**,**C**): Alpha diversity analysis of the colonic microbiota. Rarefaction curve using the number of observed operational taxonomic units (**B**) and Faith’s phylogenetic diversity (**C**). (**D**,**E**): Beta diversity analysis of colonic microbiota Unweighted UniFrac (**D**) and weighted UniFrac (**E**). N = 5 per group. OTU: operational taxonomic unit, PD: phylogenetic diversity. * *p* < 0.05.

The detailed cecal bacterial composition at the species level is shown in Table S2. Phylogenic analyses of the 16S ribosomal RNA gene revealed eight phyla in the colonic microbiota. The most predominant phylum in the colon was Firmicutes in both sham and OVX mice. The relative abundances of Actinobacteria and Proteobacteria were significantly higher in OVX mice than in sham mice (*p* = 0.008 and *p* = 0.032, respectively) (Figure 2A).

The colonic microbial diversity was compared using rarefaction analysis and Faith’s phylogenetic diversity (Figure 2B,C), and the alpha diversity was significantly lower in OVX mice than in sham mice. Comparison of beta diversities in the colon between the two groups revealed significant differences in unweighted UniFrac (Figure 2D) but not in weighted UniFrac (Figure 2E). There were no significant differences in alpha or beta diversity between sham and OVX mice in the small intestine. (Figure S3A–E).

### 3.4. Antibiotics Decreased Bone Formation but Did Not Affect Bone Mineral Density

**Figure 3:**
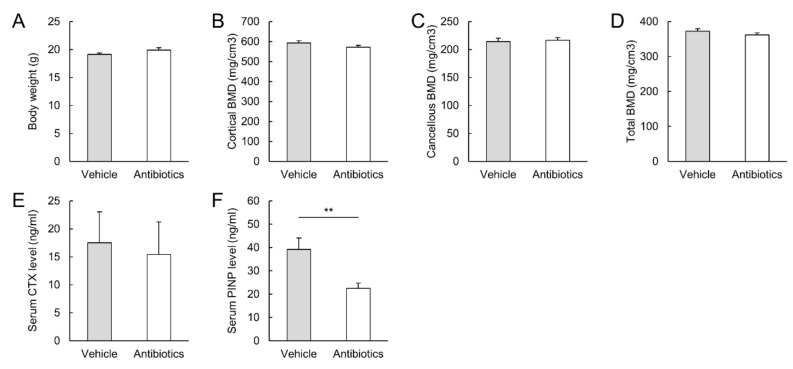
Effect of antibiotics mixture on bone homeostasis. Mice were orally administered antibiotics or vehicle for four weeks. (**A**–**F**): Body weight, tibial bone mineral density (**B**–**D**), serum bone formation marker (**E**), and serum bone resorption marker (**F**) at sacrifice. N = 7–10 per group. BMD: bone mineral density, CTX: *C*-terminal telopeptide of collagen, PINP: procollagen type I *N*-terminal propeptide. ** *p* < 0.01.

The effect of the gut microbiota on bone formation and BMD was examined after oral administration of antibiotics. Antibiotic administration did not change body weight (Figure 3A). Although tibial BMDs were not different between antibiotic-treated and vehicle-treated mice (Figure 3B–D), bone formation and resorption did not occur with antibiotic administration. Serum PINP levels were decreased to 57% after four weeks of treatment with a mixture of antibiotics compared to those in vehicle-treated mice. However, antibiotic administration did not affect serum CTX-1 levels (Figure 3E,F).

### 3.5. Fecal Microbiota Transplantation from OVX Mice to Antibiotic-Treated Non-OVX- or OVX Mice Did Not Change Bone Homeostasis

**Figure 4:**
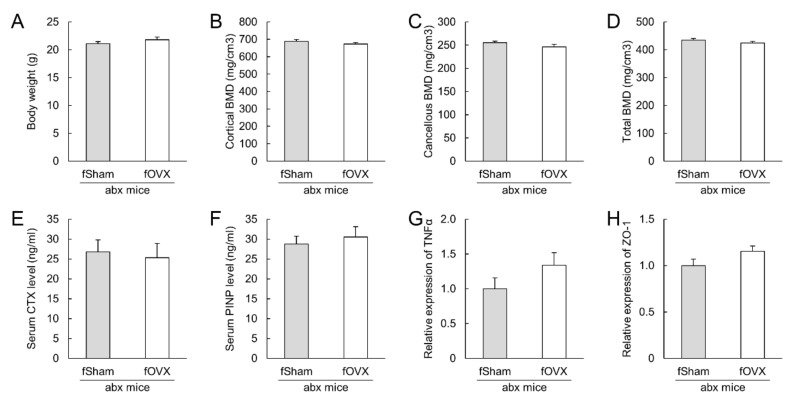
Fecal microbiota transplantation from ovariectomized (OVX)- and sham- mice to antibiotic-treated mice. Antibiotic-treated mice were orally administered cecal luminal contents of sham or OVX mice three times per week for four weeks. (**A**): Body weight at sacrifice (**B**–**D**): Tibial bone mineral density. (**E**,**F**): Serum bone formation marker (**E**) and serum bone resorption marker (**F**). (**G**,**H**): Relative mRNA expression of TNFα (**G**) and ZO-1 (**H**). N = 8–9 per group. BMD: bone mineral density, CTX: *C*-terminal telopeptide of collagen, LPS: lipopolysaccharide, PINP: procollagen type I *N*-terminal propeptide, TNFα: tumor necrosis factor alpha, ZO-1: zonula occludens-1.

**Figure 5:**
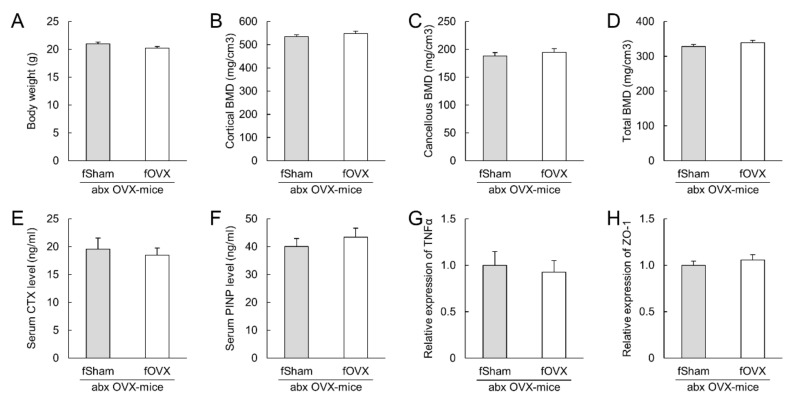
Fecal microbiota transplantation from ovariectomized and sham mice to antibiotic-treated ovariectomized mice. Antibiotic-treated mice were ovariectomized and orally administered cecal luminal contents of sham and ovariectomized mice three times per week for four weeks. (**A**–**F**): Body weight, tibial bone mineral density (**B**–**D**), serum bone formation marker (**E**), and serum bone resorption marker (**F**) at sacrifice. (**G**,**H**): Relative mRNA expression of TNFα (**G**) and ZO-1 (**H**). N = 6–7 per group. BMD: bone mineral density, CTX: *C*-terminal telopeptide of collagen, LPS: lipopolysaccharide, PINP: procollagen type I *N*-terminal propeptide, TNFα: tumor necrosis factor alpha, ZO-1: zonula occludens-1.

Cecal luminal contents collected from OVX and sham mice were transplanted into antibiotic-treated mice to investigate the role of gut microbiota. There were no significant differences in body weight, tibial BMD, or serum levels of CTX-1 or PINP levels between the two groups (Figure 4A–F). The mRNA levels of TNFα and ZO-1, which increased in OVX mice, were not changed in the colon (Figure 4G–H). Furthermore, the luminal cecal contents were also transplanted into antibiotic-treated OVX mice. Bodyweight, tibial BMD, serum bone biomarkers, and mRNA levels of TNFα and ZO-1 were not different between the two groups (Figure 5A–H).

## 4. Discussion

We found that ovariectomy decreased colonic microbial richness. However, transplantation of the gut microbiota from control mice to antibiotic-treated OVX mice did not improve BMD, and transplantation from OVX mice to antibiotic-treated mice did not worsen BMD. The administration of antibiotics to mice decreased the bone formation marker; however, BMD or the expression of the bone resorption marker were not affected. Estrogen deficiency affects the microbiota of the colon, but the effect of dysbiosis on bone homeostasis was not evident.

Similar to our results, changes in the colonic [27] or fecal [28] microbiota have previously been reported after ovariectomy. Choi et al. found a marked increase in Firmicutes in the colonic microbiota of OVX mice [27], and Cox-York et al. demonstrated increased Bacteroidetes in the feces of OVX rats [28]. Our results indicated that Actinobacteria increased in the colonic microbiota of OVX mice. Therefore, although differences in the environment across facilities may have affected changes in the gut microbiota [29], it is evident that ovariectomy causes dysbiosis.

To further elucidate the role of the gut microbiota in bone metabolism, some groups have recently discussed the effect of the gut microbiota on bone using germ-free mice. However, the results of these studies are controversial, and the specific role of the gut microbiota in maintaining bone homeostasis remains unclear. Several groups demonstrated that germ-free mice had increased bone mass compared to conventional mice, and bone was altered after colonization with the microbiota of conventional mice [11,17]. On the contrary, Quach et al. reported that microbiota reconstitution did not cause bone loss in germ-free mice [16]. There may be slight differences in experimental conditions between these studies using germ-free mice. This study used antibiotic-treated mice due to immunological defects in germ-free mice [30].

Although antibiotics do not render the mice completely germ-free, researchers have found that the use of a mixture of antibiotics can alter the acquired gut microbiota [31,32,33], as in our study. Several studies have examined the effect of antibiotic administration on bone. The reported impact of antibiotics on bone is pleiotropic and may depend on the type of antibiotic, the dose, or the duration of administration [12,34,35]. The same antibiotic may have different effects on bone depending on the duration of administration. For example, Yan et al. reported that a one-week antibiotic treatment decreased bone resorption, while a four-week antibiotic treatment decreased bone formation [12]. Our results demonstrated that commensal bacteria did not significantly affect BMD, although only serum PINP concentration was reduced by antibiotic administration. Therefore, the effect of intestinal bacteria on bone, although present, was not considered significant. Furthermore, FMT using the colonic microbiota of OVX mice did not worsen the bones of antibiotic-treated mice, whereas FMT using the colonic microbiota of vehicle-treated mice did not improve the bone of OVX mice. These results suggest that changes in the colonic microbiota do not directly affect the bones and that these changes are only a result of OVX.

Another typical method of altering the gut microbiota is the administration of probiotics. Several studies have reported that probiotic administration prevented bone loss after ovariectomy in mice by increasing anti-osteoclastogenic cytokine production [19], improving the absorption of minerals such as calcium, phosphate, and magnesium [36,37], or reducing intestinal inflammation [11]. However, this effect of probiotics on bone may not be driven by modulating the commensal microbiota composition but may be due to metabolites produced by the probiotics. To our knowledge, however, there are no reports that specific bacteria, except probiotics and FMT, have any effect on bone. Studies by other groups did not exclude the conclusion that changes in the gut microbiota do not affect bone homeostasis, as in our study.

We observed a mild elevation of serum LPS levels in OVX mice, probably due to impaired function of intestinal barriers, such as ZO-1, a tight junction-associated protein associated with intestinal inflammation caused by estrogen deficiency. Considering the harmful effects of LPS on bone [38,39,40], the increased amounts of circulating LPS could affect bone loss after ovariectomy. In the present study, FMT with luminal cecum contents of different compositions did not affect bone metabolism in wild-type or OVX mice, suggesting that the composition of the gut microbiota used for FMT did not affect bone metabolism differently. Our findings suggest that although LPS may be involved in bone metabolism, differences in LPS concentration due to differences in the composition of the gut microbiota may not be significant enough to affect bone metabolism.

This study had several limitations. First, we did not perform an FMT experiment using germ-free mice. However, several studies have reported the use of antibiotic administration to decontaminate the gut before FMT-mediated remodeling of the gut microbiota. Second, the duration of the intervention, such as antibiotic administration and FMT, might have been too short to affect the bone. However, since the observation period of four weeks is often used in other bone studies and it is expected that mice are sacrificed four weeks after surgery in experiments using OVX mice, we consider that the experimental period was appropriate. Serum bone markers, which are expected to change immediately after the intervention, did not change significantly even after four weeks, suggesting that the gut microbiota might not affect bone metabolism in a longer-term experiment. Another limitation is that we did not evaluate bone microarchitecture, which is a parameter of bone quality. Since the results of the bone marker measurement with ELISA in the FMT experiment did not show any variation, it was judged that further investigation was not necessary.

In conclusion, although ovariectomy induces dysbiosis in the colon, changes in the gut microbiota may only have a minor role in ovariectomy-induced osteoporosis.

## Data Availability

All data are available from the paper and its Supplementary Materials.

## Supplementary Materials

The following are available online at https://www.mdpi.com/article/10.3390/microorganisms9122563/s1, Figure S1: The protocols of fecal microbiota transplantation, Figure S2: The mRNA expression level of TNFα and ZO-1 in the small intestine, Figure S3: Changes in small intestinal microbiota after ovariectomy, Table S1: The PCR primers and TaqMan probes, Table S2: The cecal bacterial composition at the species level.
