# Supplementary material for: Ovariectomy-Induced Dysbiosis May Have a Minor Effect on Bone in Mice
Source: Microorganisms. 2021 Dec 10;9(12):2563. doi: 10.3390/microorganisms9122563 (PMC8708113; doi:10.3390/microorganisms9122563)
Supplement: Supplementary file 1 [file microorganisms-09-02563-s001.zip › Figures S1-S3.pdf]

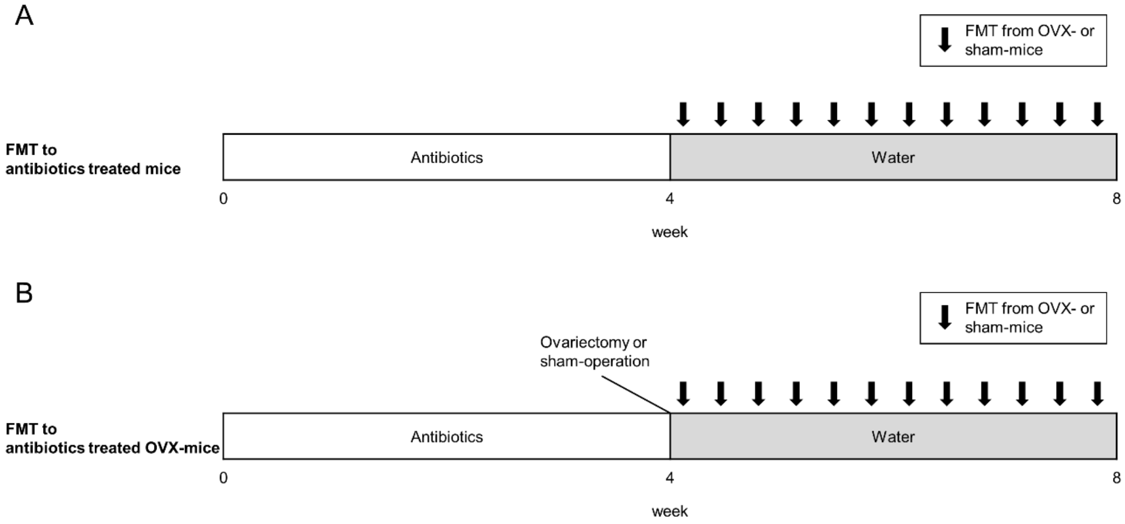

Figure S1. The protocols of fecal microbiota transplantation. Four-week-old mice were administered antibiotics for four weeks. After antibiotic treatment was completed, fecal microbiota transplantation was performed by orally administering the cecal luminal contents of sham and ovariectomized mice three times per week for four weeks (A). The ovariectomy or sham operation was performed after antibiotic treatment in preparation for the fecal microbiota experiments (B). FMT: fecal microbiota transplantation.

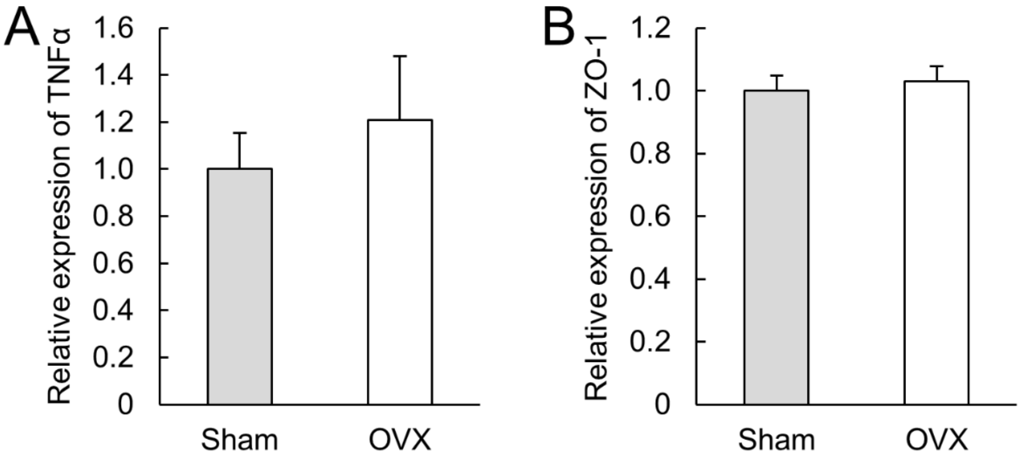

Figure S2. The mRNA expression level of TNFα and ZO-1 in the small intestine. A, B: Comparison of relative mRNA expression of TNFα (A) and ZO-1 (B) in the small intestine between sham and ovariectomized mice. N = 5 per group. TNFα: tumor necrosis factor alpha, ZO-1: zonula occludens-1.

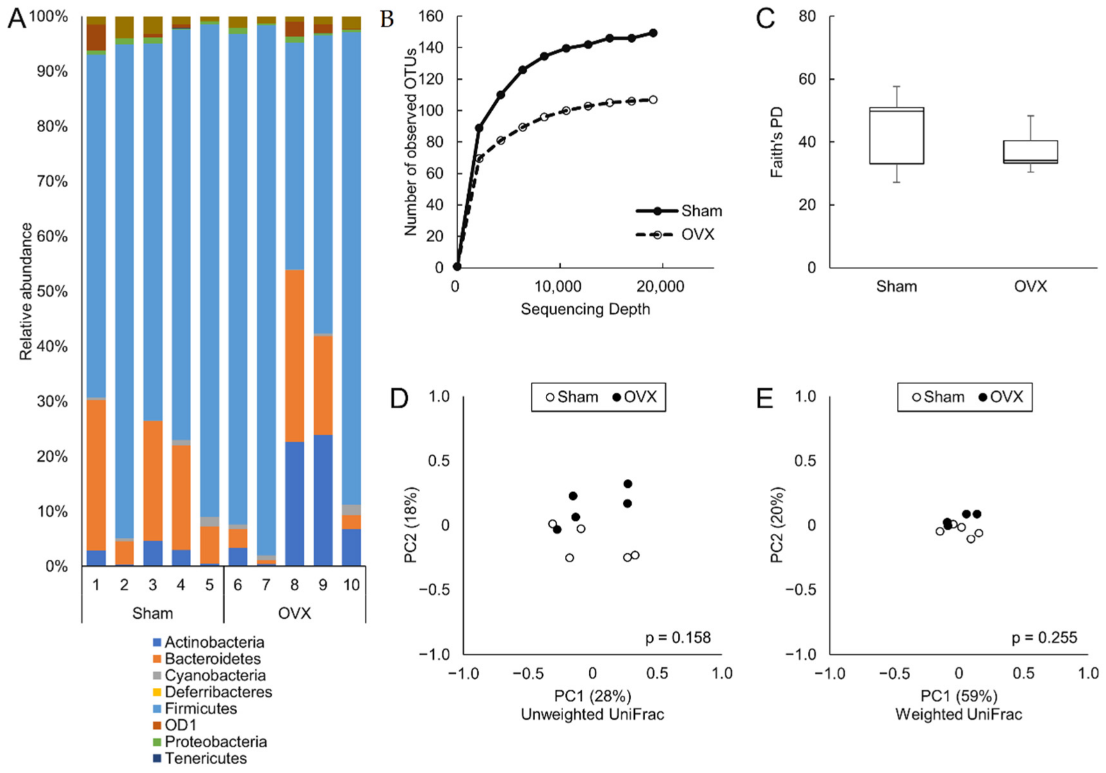

Figure S3. Changes in small intestinal microbiota after ovariectomy. A–E: Comparison of microbiota in the small intestine between sham - and ovariectomized mice. Microbial composition at the phylum level (A) Alpha diversity analysis with rarefaction curve (B) and Faith's phylogenetic diversity (PD) (C). Beta diversity analysis with unweighted (D) and weighted UniFrac (E). N = 5 per group. OTU: operational taxonomic units, PD: phylogenetic diversity.
